# Multifunctional charge transfer plasmon resonance sensors

**DOI:** 10.1515/nanoph-2023-0196

**Published:** 2023-05-17

**Authors:** Alemayehu Nana Koya, Wei Li

**Affiliations:** GPL Photonics Laboratory, State Key Laboratory of Luminescence and Applications, Changchun Institute of Optics, Fine Mechanics and Physics, Chinese Academy of Sciences, Changchun, Jilin, 130033, P. R. China

**Keywords:** charge transfer plasmons, localized surface plasmons, plasmonic sensors, single-molecule conductance sensing

## Abstract

Charge transfer plasmon (CTP) modes arise when metallic nanoparticle dimers are connected by a conductive junction. This offers a unique opportunity to explore electron transport at optical frequencies as well as to attain narrow plasmon resonances that can be broadly modulated from visible to IR regimes, implying their potentials for applications in single-molecule electronics and sensing. This article showcases recent developments in theory and applications of charge transfer plasmon resonances (CTPRs) in various configurations of conductively linked plasmonic nanodimers. In particular, we give a due attention to exploiting ultratunable spectral features of charge transfer plasmon resonances for developing multifunctional plasmonic sensors for bulk, surface, gas and molecular sensing applications. We also discuss the implications of the charge and energy transfer between two plasmonic nanoparticles linked by sub-nanometer thick self-assembled monolayers for single-molecule conductance sensing and molecular electronics. In addition to the well-established plasmonic sensing schemes based on propagating and localized surface plasmon resonances, charge transfer plasmon resonance sensors may open up a new route in efforts to develop multifunctional sensing technologies.

## 1 Introduction

The interaction of light with nanostructured noble metals gives rise to excitation of surface plasmon resonance – a collective oscillation of free electrons in the conduction band of metallic nanostructures [1]. Plasmon resonance is associated with exotic optical phenomena such as strong resonances, hugely enhanced and extremely confined near fields, and photothermal heating [2, 3], which have been widely exploited for different applications including enhanced spectroscopy [4], photocatalysis [5], optical trapping [6], single-molecule studies [7], and sensing [8]. The coherent excitation of surface plasmons can take various forms like propagating surface plasmon resonance (SPR) at metal-dielectric interface, localized surface plasmon resonance (LSPR) in metallic nanoparticles, or charge transfer plasmon resonance (CTPR) in connected metallic nanostructures [9]. These resonance properties of metallic nanostructures are highly dependent on the size, shape, configuration, material composition of the nanostructures, and the refractive index of surrounding medium [10].

In particular, the charge transfer plasmon modes that arise in conductively bridged plasmonic nanoparticles can be broadly tuned from visible to near-infrared regime by manipulating the nanojunction conductance [11, 12]. The emergence of these low-energy modes has been observed in several configurations including metallic nanodimers [13], nanoshells [14], and particle nanochains [15]. One can also observe CTP-like modes in nearly touching plasmonic nanodimers having atomic scale inter-particle separations [16, 17]. With actively tunable plasmon resonances and versatile geometries, conductively linked plasmonic nanosystems provide a unique opportunity to study electron transport at optical frequencies, implying their potentials for accelerating development of ultrafast optoelectronic devices and ultrasensitive sensors [11]. As a result, charge transfer plasmon modes have been extensively explored by a number of researchers with the aim of understanding the underlying physics of such modes and exploiting their potentials for various applications [18, 19].

On the other hand, the plasmonic sensing schemes developed so far have been solely based on the SPR and LSPR [20–22]. In conductively linked plasmonic nanosystems, one can observe multiple modes including bonding dimer plasmon (BDP) type mode, ultratunable CTP mode, and dipole-CTP hybrid mode [23]. Given interesting spectral signatures of the charge transfer plasmon resonances, high quality factor of the dipole-CTP hybrid mode, and versatile geometric parameters of linked plasmonic nanostructures, the sensing technology can benefit a lot from charge transfer plasmon resonance. In fact, the CTPR-based sensing principle is somewhat similar to that of the LSPR-based sensing, as both schemes rely on the plasmonic responses of metallic nanoparticles. However, the former has versatile geometries with facile recognition of target molecules and broadly tunable resonances characterized by narrow spectral lines. Compared to other hybrid plasmon modes (like bonding dimer plasmon and screened bonding dimer plasmon modes that appear in metallic nanodimers), CTP modes have narrow line widths [24], and thus have higher sensitivity. Moreover, unlike the simple nanodimers, the unique configuration of conductively linked nanoparticle dimers provides a rare opportunity to study electron transport at optical frequencies [11], which implies their potentials for molecular conductance sensing [25].

**Figure 1:**
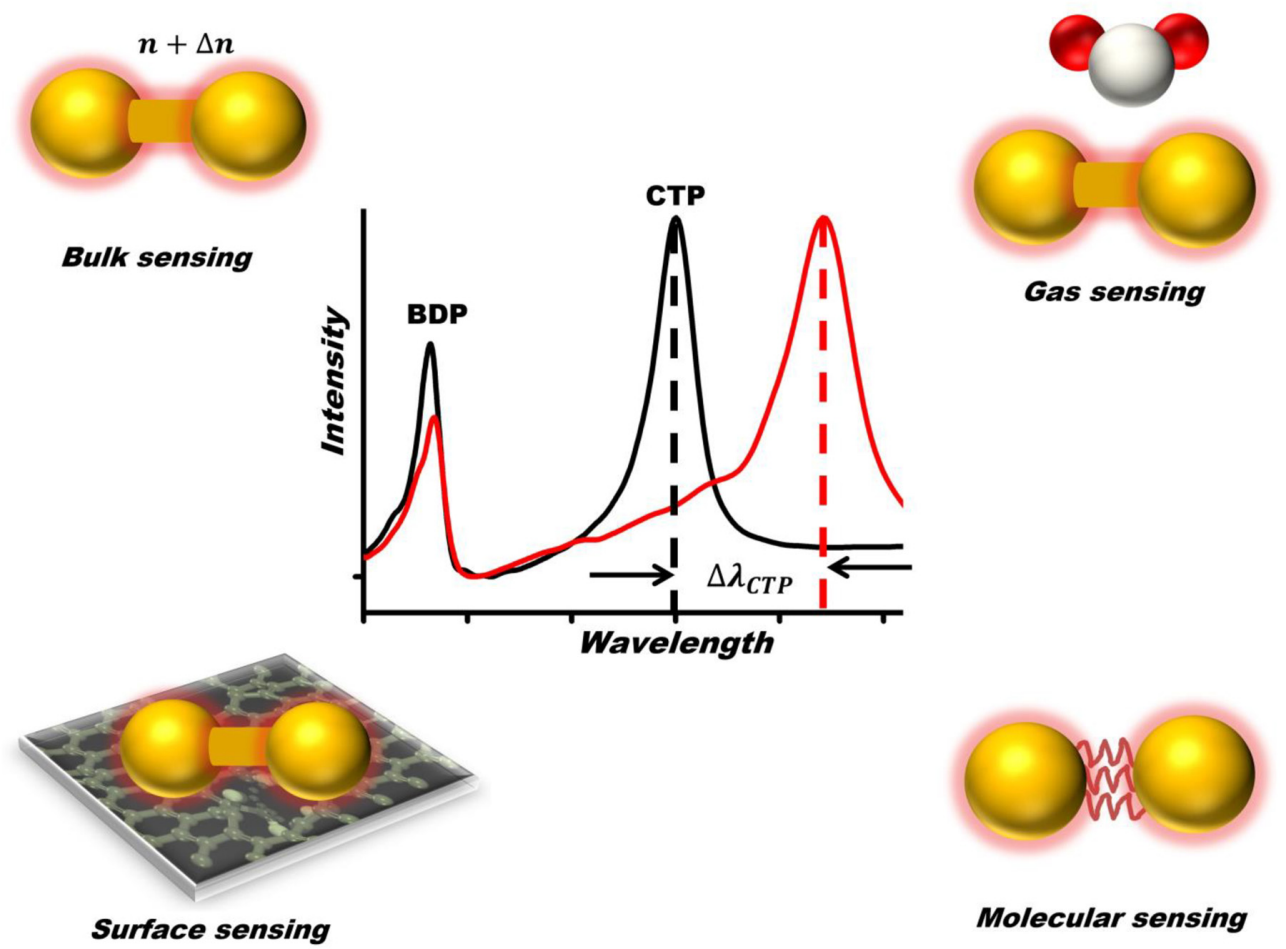
Illustration of sensing principles based on charge transfer plasmon (CTP) resonances for refractive index sensing (top left), gas sensing (top right), surface sensing (bottom left), and molecular sensing (bottom right). Middle: CTP resonance wavelength shift (Δ*λ*_CTP_) due to change in the dielectric environment of linked plasmonic nanoparticle dimer.

To take advantage of the aforementioned properties of conductively linked plasmonic nanosystems, here we overview the recent advances in the theory and applications of charge transfer plasmon resonance-based sensing. In particular, we showcase current developments in theory of charge transfer plasmons in conductively linked plasmonic nanoparticles with interparticle separations ranging from nanometer scale to atomistic regime. It is briefly discussed that the charge transfer across plasmonic junctions can be actively controlled using various materials including metals [26], palladium (Pd) nanoparticles [27], hypothetical molecules [24], DNA linkers [28], and self-assembled monolayers (SAMs) [29]. Moreover, we give a particular attention to exploiting CTP resonances for sensing applications that include bulk sensing, surface sensing, molecular sensing, and gas sensing (see Figure 1). We also showcase the potential of quantum plasmon resonances for single-molecule conductance sensing using single-molecule break junction techniques. Thus, we anticipate that these research developments in ultratunable CTPR-based sensors may lead to a paradigm shift in plasmonic sensing.

## 2 Theory of charge transfer plasmon resonances

**Figure 2:**
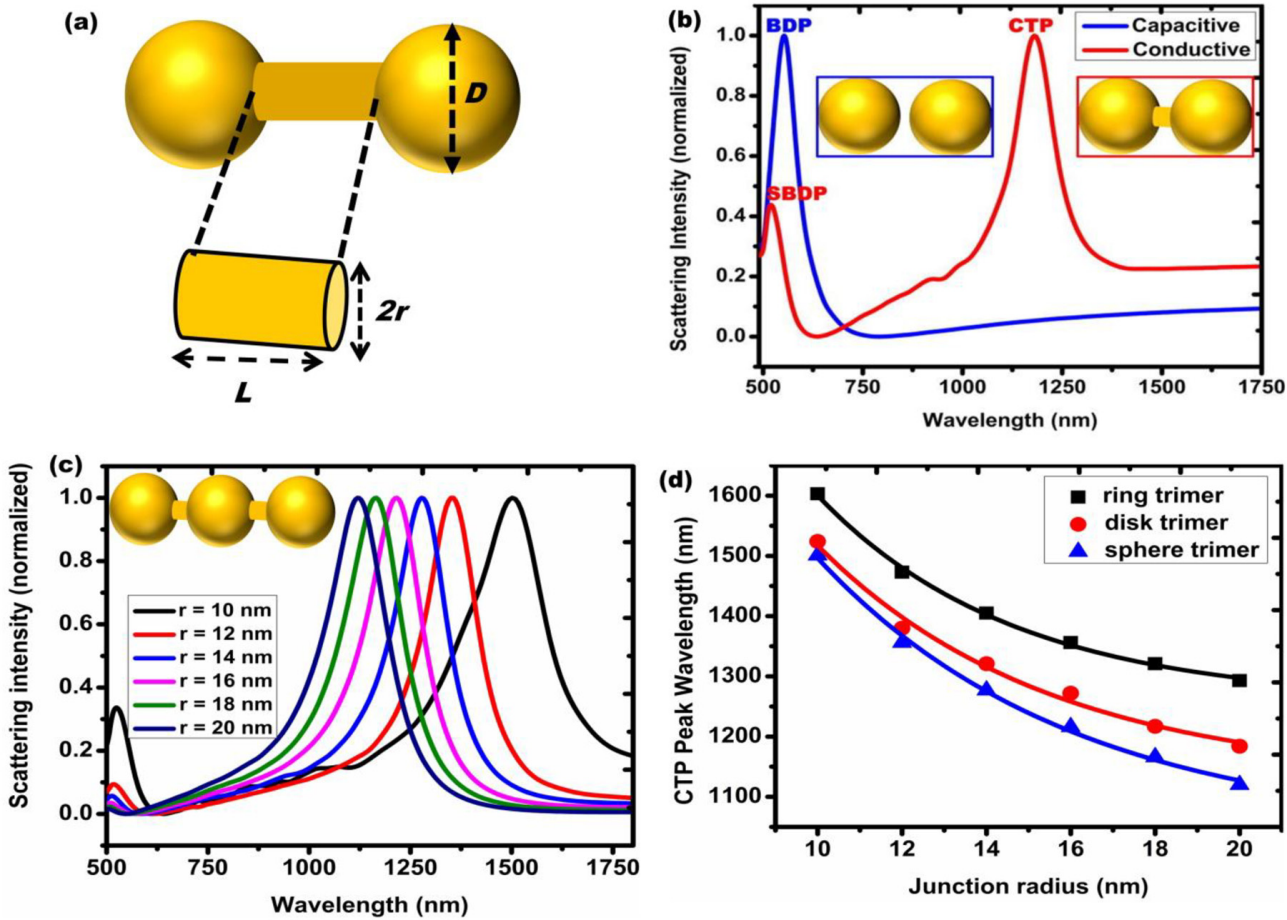
Spectral characteristics of charge transfer plasmon resonances in conductively linked plasmonic (here Au) nanoparticles. (a) Schematic illustration of geometric parameters that define CTP resonances in conductively linked plasmonic nanoparticles. These include nanoparticle size *D*, junction length *L* and radius *r*. (b) Spectral signature of the charge transfer plasmon resonance of connected Au nanoparticles. The CTP mode appears substantially at lower energies compared to other plasmon modes like screened bonding dimer plasmon (SBDP) mode of bridged nanodimer or bonding dimer plasmon (BDP) mode of unbridged nanodimer. Reproduced with permission from [18]. Copyright 2017 AIP Publishing. (c) Tuning CTP resonance of linked Au nanotrimer by changing junction conductance through its geometric parameter (here radius of cylindrical linker). (d) CTP spectral shift of conductively linked Au trimers as a function of junction radius for different nanoparticle shapes. Reproduced with permission from [12] Copyright 2016 AIP Publishing.

The electromagnetic coupling of plasmonic resonances in interacting metallic nanoparticles offers the opportunity to excite exotic plasmonic modes like charge transfer plasmon resonances in conductively linked metallic nanoparticles. For a nanoparticle dimer connected by conductive junction with nanometer scale length and thickness, one can describe the electron transport through the junction using classical electrodynamics. Generally, the conductance *G* of cylindrical nanojunction having radius *r* and length *L* (see Figure 2a) can be expressed as [12].

(1) $$G=\frac{\sigma \pi r^{2}}{L}$$

where *σ* is the conductivity of the nanojunction. Nevertheless, the onset of CTP modes in conductively linked plasmonic nanoparticles depends not only on the junction conductance as shown in Equation (1) but also on the size of the linked nanoparticles. One can determine the conductance threshold for the onset of CTP resonance in linked plasmonic nanosystems with [30, 31].

(2) $$G_{\text{CTP}}=\frac{c}{8\lambda_{\text{CTP}}}\frac{D^{2}}{L}$$

where *λ*_CTP_(= 2*πc*/*ω*_CTP_) is the resonance wavelength of charge transfer plasmon and *D* is the diameter of spherical nanoparticle as illustrated in Figure 2a. When the minimum requirement implied in Equation (2) is met, one can observe CTP resonance, which appears at longer wavelength compared to the bonding dimer plasmon (BDP) mode (Figure 2b).

Extending this fact to more complex geometries and various shapes, Koya and Lin developed a generalized analytical expression for charge transfer plasmon resonance shift as functions of junction radius *r* and nanoparticle shape [12, 18].

(3) $$\lambda_{\text{CTP}}(r)=\lambda_{\text{CTP}}^{0}+\frac{C}{\exp\left[\frac{r-r_{0}}{t}\right]}$$

where $\lambda_{\mathrm{CTP}}^{0}$ is the CTP resonance wavelength at the smallest junction radius (*r* = 10 nm in this case), and *C*, *r*_*0*_ and *t* are shape-specific fitting parameters provided in ref. [12]. As implied in Equation (3), increasing the junction radius leads to a blueshift of the CTP wavelength (Figure 2c). As shown in Figure 2d, the CTP spectral shift depends not only on the nanojunction conductance but also on the size and shape of the nanoparticles linked by the junction. This is attributed to the excitation of sub-radiant and super-radiant plasmon modes that arise in complex nanostructures like nanoring and nanodisk dimers [32, 33]. Nevertheless, Equation (3) holds true only for CTP resonance shift of linked Au nanoparticles and it may not be applied for all linked metallic nanodimers, as the equation does not incorporate the intrinsic dielectric properties of materials.

Generally, the resonance shift of coupled plasmonic nanodimers having nanometer scale separations can be safely modeled with the classical electrodynamics [34, 35]. And the spectral line widths of CTP resonances is narrower than other dimeric modes like bonding dimer plasmon (BDP) or screened bonding dimer plasmon (SBDP) modes that appear in metallic nanodimers. It was experimentally demonstrated that, at optimum junction geometry (*r* = 15 nm), the CTP and BDP resonances in linked Au nanodisk dimers could yield spectral line widths of about 0.094 and 0.333 eV, respectively [11], implying that these ultranarrow CTP resonances can be exploited for sensing applications [24]. However, with the increase in nanowire radius and the concurrent blue-shift of the CTP to higher energies, the line width of the CTP becomes broader as a result of increased radiation damping [11].

**Figure 3:**
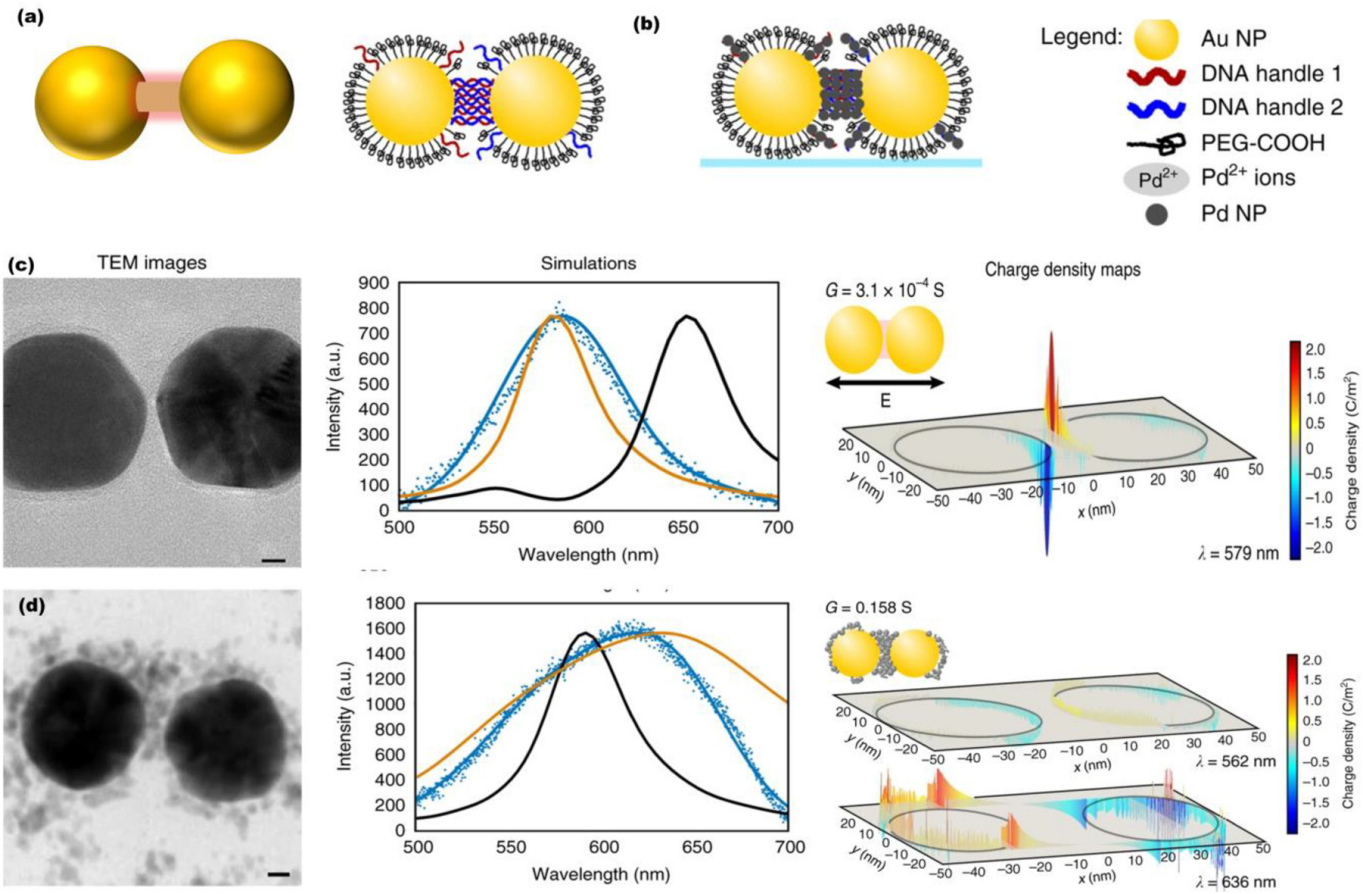
Active control of charge transfer in plasmonic nanodimers linked by molecular junctions. Top: Schematic illustrations of (a) quantum corrected model mimicing electrron tunneling through a molecular linker and (b) Au nanoparticle dimer functionalized with DNA molecules incorporated into a monolayer of PEG-COOH (left) and integrated with Pd nanoparticless (right). Bottom: TEM analysis, spectroscopy and charge density maps of Pd nanoparticles in plasmonic nanodimers with gap width of (c) 2 nm and (d) 18 nm. Reproduced under terms of the CC-BY license from [27]. Copyright 2018, The Authors, published by Nature Publishing Group.

On the other hand, when the size of interparticle separation enters to sub-nanometer scale, the classical universal scaling relationship fails to describe the spectral shift, as non-local screening and electron tunneling effects emerge in such atomic scale gaps [36]. The non-local effect is well-described by Landau damping, that is, direct excitation of electron–hole pairs in the metals by the highly confined electric fields of surface plasmons [37]. It was theoretically demonstrated that the Landau damping affects the field enhancement, effective volume, and line width of plasmon resonances in metallic nanodimers [38], hence limit their applications in sensing. Moreover, as the nanodimer gap enters subnanometer regime, apart from the plasmon-induced screening charge nonlocal effect, electron tunneling across the junction also induces a drastic change in plasmonic responses [39–41]. To describe such effects in atomic scale gaps, Esteban et al. developed quantum-corrected model (QCM), which incorporates quantum-mechanical effects within a classical electrodynamic framework [42]. QCM has been widely employed to describe charge transfer in molecularly linked plasmonic nanodimers with sub-nm gaps, where one can actively control the charge transfer across the conductive junction by modeling its conductance with various materials such as Pd and DNA molecules (see Figure 3a) [43, 44].

In this regard, Lerch and Reinhard explored the role of DNA mediated charge transfer in plasmonic nanodimers, experimentally demonstrating DNA mediated optical tunneling in Au nanodimers with separations as small as 2.8 nm [28]. In related work, they investigated the effect of interstitial nanoparticles on the distance-dependent plasmon coupling between DNA-connected Au nanoparticles [27]. In particular, they studied the effect of increasing Pd nanoparticles intragap density on the optical response of DNA-tethered plasmonic nanodimers (see Figure 3b). They demonstrated that high Pd densities in the inter-particle gap increases the gap conductance and induces the transition from capacitive coupling to conductive coupling (Figure 3c & d). Pd was chosen since its cations are best known to bind to DNA and Pd–Au heterostructures have interesting implications for sensing applications [27]. Further details on the underlying physics of charge transfer plasmons can be found in the recent reviews on the topic [18, 19].

## 3 Bulk and surface sensing characteristics of CTP resonances

The principle of sensing with charge transfer plasmon resonances was first reported by Perez-Gonzalez et al., which theoretically demonstrated that plasmonic nanodimers linked by narrower junctions are more sensitive to the embedding medium as the conductance increases [31]. Subsequently, they also demonstrated that sharp and ultratunable spectral features of CTP resonances in linked nanodimers can be used for bulk sensing, where they reported high performing CTPR-based sensor with figure-of-merit (FOM) as high as 12.4 [24]. Since then, a number of theoretical works have been reported including the most recent one by Dana et al. in which they optimized conductively linked asymmetric Au nanodimer for refractive index sensing [26]. They found that, compared to linked symmetric nanodimers, asymmetric nanodimers have higher bulk sensing capability (FOM = 10.88) owing to the excitation of hybridized low-loss modes, like Fano resonances, in such systems [8, 45].

**Figure 4:**
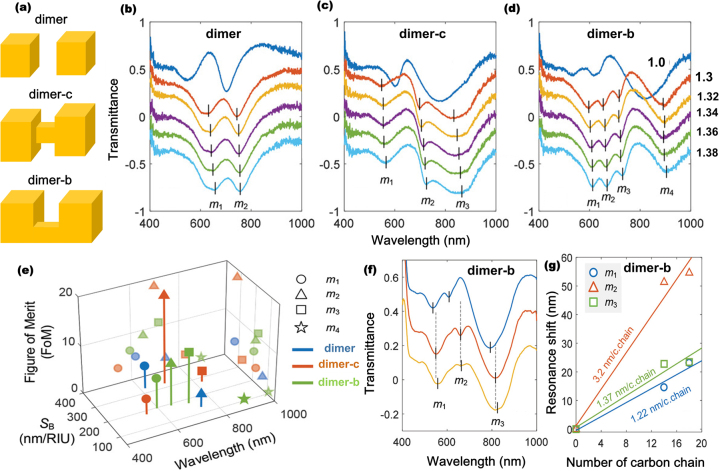
Charge transfer plasmon resonance-based bulk and surface sensing. (a) Schematic of capacitively coupled sub-5 nm dimer (dimer) and conductively coupled dimers with center (dimer-c) and bottom (dimer-b) bridges of 40 nm width. Each cubic Au nanodimer has a height of *h* = 150 nm. Transmission spectra shift as a function of refractive index for (b) dimer, (c) dimer-c, and (d) dimer-b nanostructures. (e) Bulk sensing performance of dimer, dimer-c, and dimer-b structures based on cladding index *n*_cl_ = 1.3. (f) Transmission spectra and (g) surface sensitivity characteristics of dimer-b as a function of number of carbon chains. Reproduced with permission from [23]. Copyright 2021, Wiley-VCH.

Another notable work on CTPR-based sensing has been reported by Tobing et al. that demonstrated the emergence of dipole, CTP and hybridized modes in different plasmonic nanostructures including capacitively coupled dimers with sub-5 nm gap and conductively coupled dimers [23]. They designed various plasmonic nanodimers including sub-nm gap unlinked dimers as well as conductively coupled dimers connected with junction bridge positioned at the center (dimer-c) and at the bottom (dimer-b) (see Figure 4a), with the intention of exploiting such nanoarchitectures for bulk and surface sensing applications. As a result of change in the cladding refractive index from *n* = 1 to *n* = 1.3, the transmission spectra of these nanostructures can show various modes including longitudinally oscillating dimer-mode (*m*_1_) and CTP-like mode (*m*_2_) in the dimer; dimer mode (*m*_1_), hybrid dimer-CTP mode (*m*_2_), and CTP mode (*m*_3_) in dimer-c; and dimer mode (*m*_1_), hybrid dimer-CTP mode (*m*_2_), additional mode (*m*_3_), and CTP mode (*m*_4_) in dimer-b (see Figure 4b–d). In particular, it was found that the conditional emergence of a hybrid dimer-CTP mode of conductively linked dimers based on the cladding refractive index and analyte thickness can present an opportunity for highly specific surface sensing applications (see Figure 4e–g). The aforementioned and other works on charge transfer plasmon resonances imply that these versatile and actively tunable surface plasmon resonances can be exploited for detecting various samples ranging from chemicals and hazardous gas molecules to bulk materials and single molecules (see Table 1).

**Table 1:** Charge transfer plasmon resonance-based sensing with different configurations of conductively linked plasmonic nanodimers.

Dimer	Gap	Resonance	Gap	Sensitivity	FOM	Detection	Method	Ref.
configuration	size	wavelength	material	[nm/RIU]		target		
Cube dimer	5 nm	628 nm	Air	329^a^	4.8	RI	Experimental	[23]
Ring-disk dimer	20 nm	1760 nm	Au	840	10.88	RI	Theoretical	[26]
Cube dimer	15 nm	830 nm	Au	326^b^	18.4^b^	RI	Experimental	[23]
Cube dimer	15 nm	–	Au	3.2^c^	–	Carbon chains	Experimental	[23]
Sphere dimer	5 nm	839 nm	Pd	–	–	O_2_	Theoretical	[44]
Sphere dimer	1 nm	800 nm	Molecule	3154.8	12.8	RI	Theoretical	[24]
Particle-on-mirror	1 nm	665 nm	Molecule	–	–	SAMs	Experimental	[57]

^a^From two modes that can arise in cube dimer, only the dimer mode (*m*_1_) is considered in this case; ^b^These data represent sensitivity of dimer-CTP hybrid mode, which arises from the interaction between dimer mode (*m*_1_) and CTP mode (*m*_3_) as shown in Figure 4; ^c^This result denote CTP mode surface sensitivity defined as *S* = Δ*λ*/Δ*t*_*a*_, where *t*_*a*_ is analyte thickness.

## 4 Tunneling CTP resonances for molecular conductance sensing

**Figure 5:**
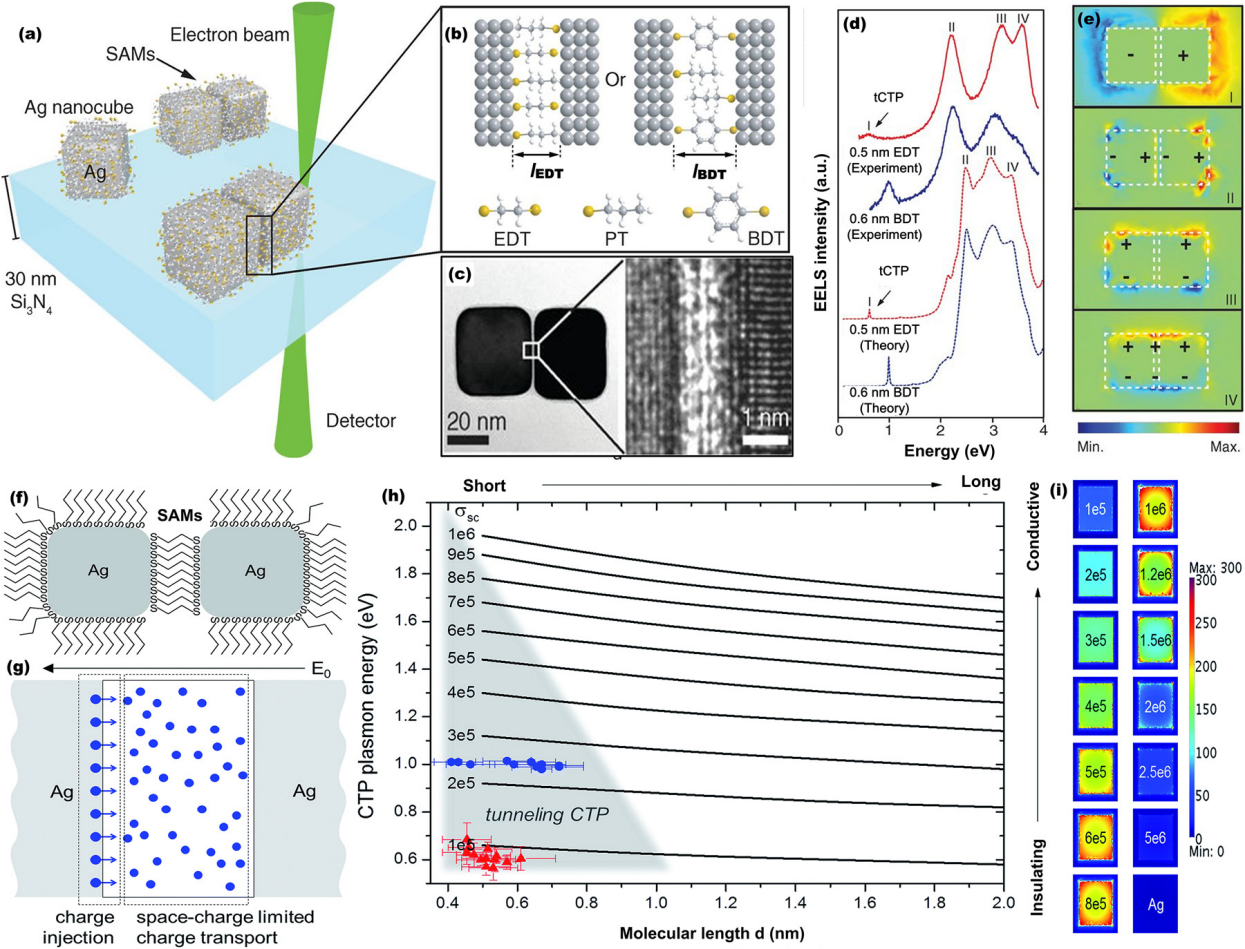
Molecular tunnel junction controlled quantum plasmon resonances in plasmonic nanodimers linked by self-assembled monolayers (SAMs). (a) Schematic illustration of the molecular tunnel junctions made of two Ag nanoparticles bridged by SAMs of EDT (1,2-ethanedithiolates) and BDT (1,4-benzenedithiolates). (b) Active control of the distance between two adjacent Ag nanoparticles through the EDT and BDT thickness. (c) A high-resolution TEM image of the Ag–SAM–Ag junction. (d) Measured EELS spectra and quantum-corrected simulations of extinction spectra of Ag–SAM–Ag nanosystem, confirming quantum tunneling directly observed as tunneling charge transfer plasmon (tCTP) peak. (e) Simulated electric near-field distributions for the designated plasmonic modes I to IV. Reproduced with permission from [29]. Copyright 2014, American Association for the Advancement of Science. (f) Schematic diagram of hybrid Ag–SAM–Ag system. (g) Space-charge corrected electromagnetic model to describe the charge transfer plasmon oscillations of the Ag–SAM–Ag junction, where negative driving field (during half cycles) induces charge transfer from left to right. (h) Constructed parameter map to correlate resonant CTP energies (eV), SAM conductivity ***σ***_***sc***_ (S/m), and SAM molecular lengths *d* (nm) in the Ag–SAM–Ag system for fixed Ag nanocube dimesnions. (i) Magnitudes of total electric field enhancements at the Ag/SAM interface for various gap conductivities at respective CTP resonant frequencies. Reproduced with permission from [54]. Copyright 2016, Royal Society of Chemistry.

Apart from the classical bulk sensing applications, quantum nature of charge and energy flow through conducting linkers is of interest for various applications including understanding the dynamics of plasmon-induced charge transfer [46–48], photocatalytic water splitting [49], switchable optical materials [50], nanoelectronic devices [51] and quantum technology [52]. In particular, the quantum plasmon resonance that appears in molecular tunnel junctions made of two plasmonic nanoresonators bridged by self-assembled monolayers (SAMs) has attracted a growing research interest owing to the opportunities it offers for nano-electronics and single-molecule sensing [53, 54]. In this regard, Tan et al. [29] reported direct observation of quantum tunneling between silver nanocube resonators bridged by a SAM made of aliphatic EDT (1,2-ethanedithiolates) and aromatic BDT (1,4-benzenedithiolates) (see Figure 5a–c). The EELS spectra recorded from junctions with SAMs of EDT and BDT reveal that three main plasmon peaks (designated as bonding dipolar plasmon mode (II), transverse corner mode (III), and transverse edge mode (IV)) can be observed along with SAM thickness-dependent, low-energy plasmon mode (I) (Figure 5d). Based on the simulation result that shows the transfer of net charge between the Ag cuboiods (see Figure 5e), this new mode is assigned as tunneling charge transfer plasmon (tCTP) mode.

In related study reported by Wu et al. [54], using the space-charge corrected electromagnetic model that treats the charge injection and charge transport separately, they numerically demonstrated establishing a one-to-one relationship between the conductivity of the SAM and the resonant energy of the tCTP modes in organic-inorganic hybrid systems made of Ag cube dimer bridged by SAMs consisting of a finite number of molecules (see Figure 5f–i). They also estimated the molecular conductance at the CTP resonant frequency for two types of SAMs, where the estimated THz conductance was about 0.2*G*_0_ per EDT molecule at 140 THz and 0.4*G*_0_ for a BDT molecule at 245 THz (where *G*_0_ = 2 e⁄h is the conductance quantum), implying that the tunneling charge transfer plasmon oscillations can be used for measuring the THz conductance of single molecules at near-infrared frequencies [54].

**Figure 6:**
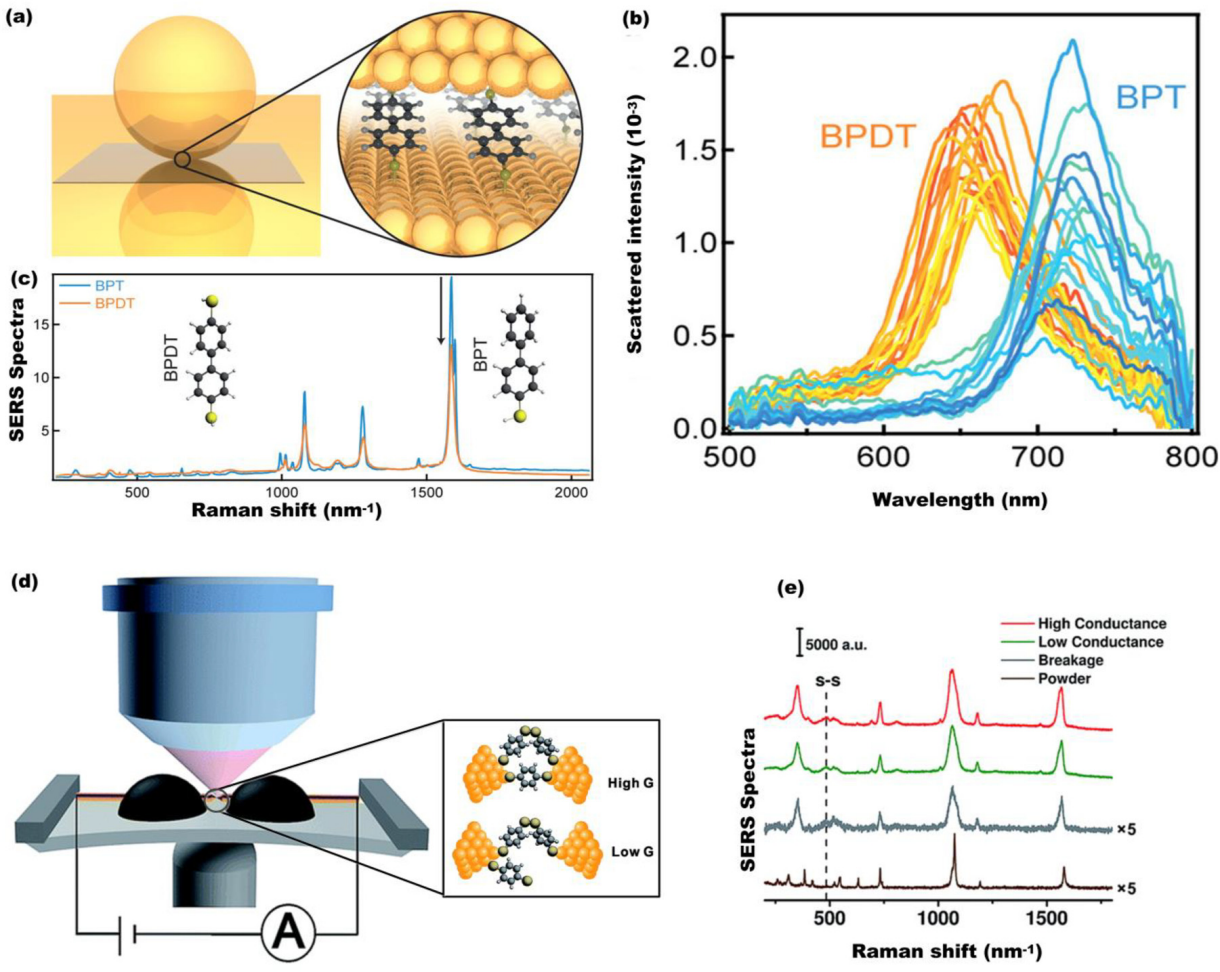
Implication of tunneling charge transfer plasmon (tCTP) for single-molecule conductance sensing. (a) Schematic of conductive (BPDT) and nonconductive (BPT) self-assembled monolayers in plasmonic junctions made of Au nanoparticle-on-mirror (NPoM) configuration. (b) Normalized scattered intensity from individual 60 nm gold nanoparticles on BPT and BPDT. (c) Average SERS spectra normalized by the number of molecules, comparing SERS intensities from BPDT and BPT. Reproduced with permission from [57]. Copyright 2014, American Chemical Society. (d) Schematic illustration of mechanically controllable break junction (MCBJ) SERS setup with molecular structures of BDT and dimeric-BDT. Inset: Hypothetical evolution of the microscopic configuration as the conductance *G* of the monolayers evolves from high conductance to low conductance. (e) SERS spectra collected when the molecular junction was mechanically controlled at the regimes of high conductance (red), low conductance (green), and breakage (grey), respectively. An ordinary Raman spectrum of BDT powder (brown) is displayed for comparison. Reproduced with permission from [59]. Copyright 2018, Royal Society of Chemistry.

These and other studies on control of charge and energy transfer properties of single-molecule junctions (SMJs) have inspired enormous research interest directing toward single-molecule sensing with the ultimate goal of developing single-molecule opto-electronic devices [55, 56]. To this end, Baumberg et al. explored molecular junction conductance of plasmonic nanodimer comprised of Au nanoparticle-on-mirror (NPoM) with actively controlled self-assembled monolayer conductive junction as small as 1 nm thick (see Figure 6a) [57]. They experimentally demonstrated about 50 nm blueshift of CTP as the junction conductance changes from insulating BPT to conductive BPDT (biphenyl-4,4-dithiol) (see Figure 6b). Using the LCR model, they also derived a simple analytical description for the blueshifted screened plasmon resonance wavelength shift as $\lambda_{screened}=\lambda_{0}/\left(1+4\varepsilon_{d}\omega_{L}^{2}/\omega_{p}^{2}\right)$ , where $\omega_{L}\left(=1/\sqrt{L_{g}C_{s}}\right)$ is inductive coupling, *ω*_*p*_(= 2*πc*/*λ*_*p*_) is plasma frequency and *λ*_*0*_ is unscreened plasmon mode of the NPoM [57, 58]. They demonstrated the conductance of 0.17*G*_0_ per BPDT molecule and a total conductance across the junction of about 30*G*_0_. By using the number of molecules determined from the conductance-induced blue-shifts, they also obtained surface-enhanced Raman spectroscopy (SERS) of both BPT and BPDT with intensities normalized by the number of molecules (Figure 6c). Similarly, Zhang et al. applied a combination of mechanically controllable break junction (MCBJ) and *in situ* SERS methods to investigate single-molecule conductance of prototypical benzene-1,4-dithiol junctions (Figure 6d & e) [59]. Plasmon-enhanced break-junction (PEBJ) has also been widely employed for trapping and manipulation of single-molecules with sizes ranging from 2 nm to 1 nm [60, 61]. In particular, Zeng et al. demonstrated direct trapping and *in situ* sensing of single molecules with sizes down to ∼5  Å in solution by employing an adjustable plasmonic optical nanogap and single-molecule conductance measurement [62]. These sophisticated works by leading researchers imply that strongly coupled and conductively linked plasmonic nanodimers can contribute to understanding molecular conductance sensing.

## 5 Concluding remarks

In this article, we have overviewed recent developments in charge transfer plasmon resonance-based sensing. When plasmonic nanoparticles are linked by conductive junction, electron flow can take place across the junction, which leads to generation of charge transfer plasmon mode when a minimum conductance threshold is met. These modes are found to be extremely tunable and they have relatively narrow spectral signature, implying their potentials for sensing applications. As a result, CTP has been focus of intense theoretical and experimental research interest for the last ten years [11, 13, 18, 27, 30].

In principle, one can broadly tune the CTP resonances of linked plasmonic nanoparticles by controlling the nanojunction conductance as well as nanoparticle size and shape. And the underlying physics of charge flow across conductive junction are described with classical electrodynamics and quantum mechanical principles. The spectral shift of charge transfer plasmon resonance in linked nanoparticles with nanometer scale inter-particle separation can be safely described using the classical universal scaling model [34]. However, when the inter-particle distance enters to the sub-nanometer regime where the non-local screening and electron tunneling effects are prominent, quantum-corrected model is employed [42]. The charge transfer in molecularly linked plasmonic nanodimers can be actively modulated by controlling the junction conductance using self-assembled monolayers, DNA molecules, and Pd nanoparticles, implying their potentials for molecular sensing and switchable opto-electronics.

In this regard, we have explored the sensing capability of CTP and alike modes that arise in strongly coupled and conductively linked plasmonic nanosystems of various configurations. In particular, we have discussed the physical principles and active tuning mechanisms of charge transfer plasmons with possible applications in bulk, surface, and molecular sensing. Beyond biosensing applications, these ultratunable plasmonic modes can also be employed for sensitive and robust detection of gases and chemical reactions [44, 63, 64]. We have also showcased recent theoretical and experimental developments in quantum plasmon resonance that appears in molecular tunnel junctions made of two plasmonic nanoresonators bridged by self-assembled monolayers and their implications for single-molecule sensing.

Finally, we would like to reiterate the fact that conductively linked plasmonic nanosystems have versatile geometries, amenable recognition of target molecules and broadly tunable plasmon resonances with ultranarrow line widths – all indicating that these structures can be employed for sensitive detection of various analytes. Moreover, the unique configuration of linked nanoparticle dimers provides a rare opportunity to study electron transport at optical frequencies, which can be exploited for molecular conductance sensing. Thus, we anticipate that linked plasmonic nanodimers with broadly tunable CTP modes can be ideal candidates for development of ultrasensitive multifunctional plasmonic sensing technologies [65, 66] and ultrafast opto-electronic nanodevices [67, 68].

## Supplementary Material

Supplementary Material Details

Supplementary Material Details

Supplementary Material Details

Supplementary Material Details

Supplementary Material Details

Supplementary Material Details

Supplementary Material Details

Supplementary Material Details
