# Supplementary material for: Multifunctional charge transfer plasmon resonance sensors
Source: Nanophotonics. 2023 May 17;12(12):2103–13. doi: 10.1515/nanoph-2023-0196 (PMC11501418; doi:10.1515/nanoph-2023-0196)
Supplement: Supplementary file 2 — Supplementary Material Details [file j_nanoph-2023-0196_suppl_002.pdf]

AIP PUBLISHING LICENSE
TERMS AND CONDITIONS

Apr 22, 2023

This Agreement between Prof. Alemayehu Nana Koya ("You") and AIP Publishing ("AIP Publishing") consists of your license details and the terms and conditions provided by AIP Publishing and Copyright Clearance Center.

License Number	5534181200836
License date	Apr 22, 2023
Licensed Content Publisher	AIP Publishing
Licensed Content Publication	Journal of Applied Physics
Licensed Content Title	Bonding and charge transfer plasmons of conductively bridged nanoparticles: The effects of junction conductance and nanoparticle morphology
Licensed Content Author	Koya, Alemayehu Nana; Lin, Jingquan
Licensed Content Date	Sep 2, 2016
Licensed Content Volume	120
Licensed Content Issue	9
Type of Use	Journal/Magazine

Requestor type	STM-Publisher
STM Publisher	De Gruyter
Format	Print and electronic
Portion	Figure/Table
Number of figures/tables	1
Will you be translating?	No
Title of new article	Multifunctional charge transfer plasmon sensors: new approach based on ultratunable plasmon resonances
Lead author	Alemayehu Nana Koya
Title of targeted journal	Nanophotonics
Publisher	De Gruyter
Expected publication date	Jun 2023
Portions	Figure 6
Requestor Location	Prof. Alemayehu Nana Koya<br>77 Yingkou Road<br><br>Changchun, Jilin 130033<br>China<br>Attn: Prof. Alemayehu Nana Koya
Total	0.00 USD

Terms and Conditions

AIP Publishing -- Terms and Conditions: Permissions Uses

AIP Publishing hereby grants to you the non-exclusive right and license to use and/or distribute the Material according to the use specified in your order, on a one-time basis, for the specified term, with a maximum distribution equal to the number that you have ordered. Any links or other content accompanying the Material are not the subject of this license.

1. You agree to include the following copyright and permission notice with the reproduction of the Material:"Reprinted from [FULL CITATION], with the permission of AIP Publishing." For an article, the credit line and permission notice must be printed on the first page of the article or book chapter. For photographs, covers, or tables, the notice may appear with the Material, in a footnote, or in the reference list.
2. If you have licensed reuse of a figure, photograph, cover, or table, it is your responsibility to ensure that the material is original to AIP Publishing and does not contain the copyright of another entity, and that the copyright notice of the figure, photograph, cover, or table does not indicate that it was reprinted by AIP Publishing, with permission, from another source. Under no circumstances does AIP Publishing purport or intend to grant permission to reuse material to which it does not hold appropriate rights.
   You may not alter or modify the Material in any manner. You may translate the Material into another language only if you have licensed translation rights. You may not use the Material for promotional purposes.
3. The foregoing license shall not take effect unless and until AIP Publishing or its agent, Copyright Clearance Center, receives the Payment in accordance with Copyright Clearance Center Billing and Payment Terms and Conditions, which are incorporated herein by reference.
4. AIP Publishing or Copyright Clearance Center may, within two business days of granting this license, revoke the license for any reason whatsoever, with a full refund payable to you. Should you violate the terms of this license at any time, AIP Publishing, or Copyright Clearance Center may revoke the license with no refund to you. Notice of such revocation will be made using the contact information provided by you. Failure to receive such notice will not nullify the revocation.
5. AIP Publishing makes no representations or warranties with respect to the Material. You agree to indemnify and hold harmless AIP Publishing, and their officers, directors, employees or agents from and against any and all claims arising out of your use of the Material other than as specifically authorized herein.
6. The permission granted herein is personal to you and is not transferable or assignable without the prior written permission of AIP Publishing. This license may not be amended except in a writing signed by the party to be charged.
7. If purchase orders, acknowledgments or check endorsements are issued on any forms containing terms and conditions which are inconsistent with these provisions, such inconsistent terms and conditions shall be of no force and effect. This document, including the CCC Billing and Payment Terms and Conditions, shall be the entire agreement between the parties relating to the subject matter hereof.

This Agreement shall be governed by and construed in accordance with the laws of the State of New York. Both parties hereby submit to the jurisdiction of the courts of New York County for purposes of resolving any disputes that may arise hereunder.

V1.2

Questions? customercare@copyright.com.
