# Supplementary material for: Multifunctional charge transfer plasmon resonance sensors
Source: Nanophotonics. 2023 May 17;12(12):2103–13. doi: 10.1515/nanoph-2023-0196 (PMC11501418; doi:10.1515/nanoph-2023-0196)
Supplement: Supplementary file 3 — Supplementary Material Details [file j_nanoph-2023-0196_suppl_003.pdf]

RightsLink

Help Live Chat

SPRINGER NATURE

Effect of interstitial palladium on plasmon-driven charge transfer in nanoparticle dimers

Author: Sarah Lerch et al

Publication: Nature Communications

Publisher: Springer Nature

Date: Apr 23, 2018

Copyright © 2018, The Author(s)

Creative Commons

This is an open access article distributed under the terms of the Creative Commons CC BY license, which permits unrestricted use, distribution, and reproduction in any medium, provided the original work is properly cited.

You are not required to obtain permission to reuse this article.

To request permission for a type of use not listed, please contact Springer Nature

© 2023 Copyright - All Rights Reserved | Copyright Clearance Center, Inc. | Privacy statement | Data Security and Privacy | For California Residents | Terms and ConditionsComments? We would like to hear from you. E-mail us at customercare@copyright.com
