# Supplementary material for: Multifunctional charge transfer plasmon resonance sensors
Source: Nanophotonics. 2023 May 17;12(12):2103–13. doi: 10.1515/nanoph-2023-0196 (PMC11501418; doi:10.1515/nanoph-2023-0196)
Supplement: Supplementary file 7 — Supplementary Material Details [file j_nanoph-2023-0196_suppl_007.pdf]

RightsLink

You have been directed to this webpage as a result of the type of license signed between the author and the American Chemical Society that provides users with some different terms of use.

Open Access licenses

If the article is open access and uses a Creative Commons public use license, e.g., CC-BY or CC-BY-NC-ND, rights and permissions questions should be directed to Creative Commons. Contact information can be found on their website at https://creativecommons.org/about/contact

If the article is open access and does not use a Creative Commons license, it is subject to the terms of an ACS public use license. For rights and permissions questions about ACS public use licenses, contact support@services.acs.org with your request, including the following information:

- A link to the ACS article from which you wish to reuse content
- The portion of content you wish to reuse (e.g., number of figures, entire article for thesis)
- A description of where the content will be reused (e.g., name of journal, book title, thesis)

Public Domain and Crown Copyright, and Other Articles:

- **For articles in the public domain:**
  Check the copyright notice that appears with the abstract or on the first page of the article/chapter.
  An article is in the public domain if all of the authors of the article worked for the U.S. Government when the article was written. Requesters who want to use material that appeared in articles that are in the public domain do not need permission if the notice on the article/chapter states, “Not subject to U.S. Copyright,” and credit to another source does not appear in the figure/table caption, if you are using figures/tables from the article.

- **For articles subject to Crown copyright:**
  Check the notice that appears with the abstract or on the first page of the article/chapter.
  If all of the authors of a requested article worked for the governments of Australia, Canada, or the United Kingdom when the article was written, a Crown copyright notice appears. Write directly to the authors of the article for permission because the authors' countries own copyright to the article.

- **For other articles not recognized by RightsLink or the ACS article delivery system:**
  If the solutions above do not apply, it's possible the error is with the article delivery system.
  Send your request directly to the ACS Copyright Office at copyright@acs.org. Send a permission request that includes a complete reference citation, the amount of material you want to use from the article/chapter (such as the specific ACS figure numbers), and where you plan to reuse the ACS material. Include your complete mailing address, your 24-hour fax number, and the specific deadline by which you need to receive our response.

Inquiries about the public domain and crown copyright should be addressed to copyright@acs.org.
